# Supplementary material for: Non-Invasive Brain Stimulation Effects on Biomarkers of Tryptophan Metabolism: A Scoping Review and Meta-Analysis
Source: Int J Mol Sci. 2022 Aug 26;23(17):9692. doi: 10.3390/ijms23179692 (PMC9456364; doi:10.3390/ijms23179692)
Supplement: Supplementary file 1 [file ijms-23-09692-s001.zip › Figure S1 Biomarkers by individual regions.pdf]

(a) Brain tissue (cortex) in preclinical studies

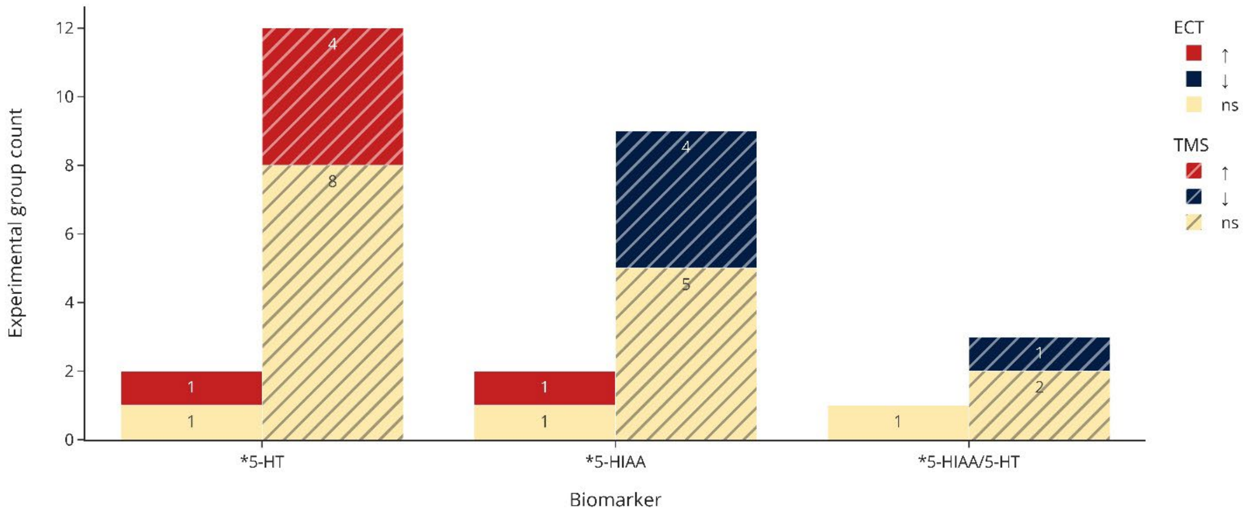

(b) Brain tissue (caudate nucleus) in preclinical studies

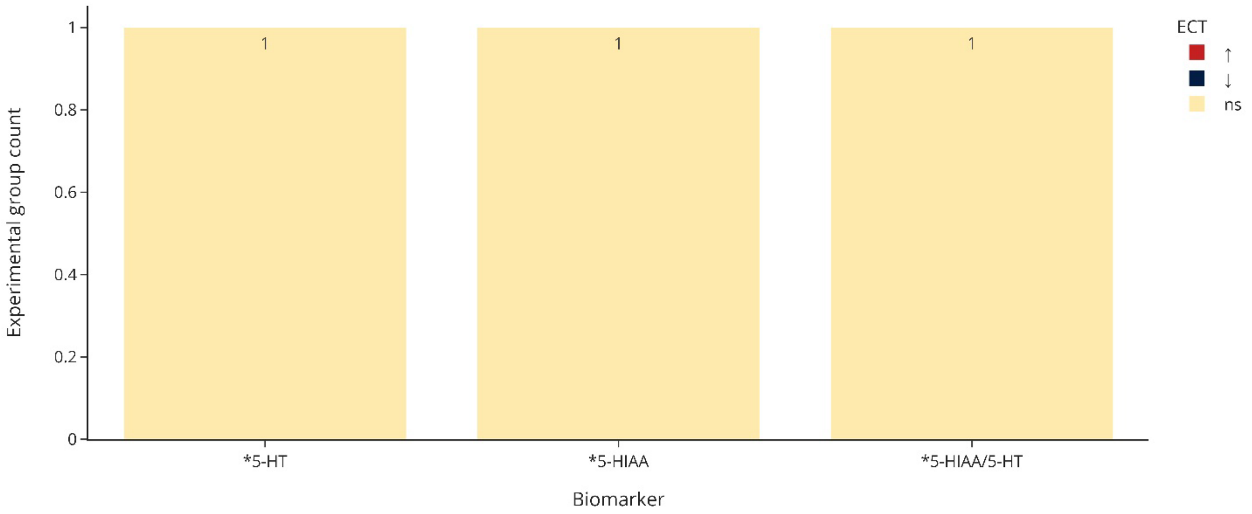

(c) Brain tissue (hippocampus) in preclinical studies

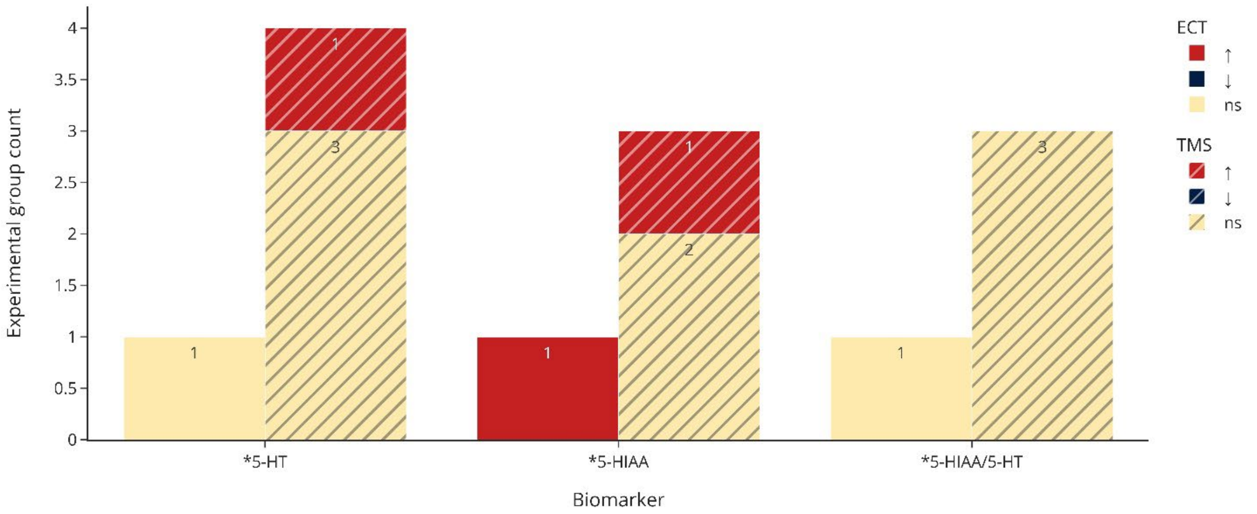

(d) Brain tissue (hypothalamus) in preclinical studies

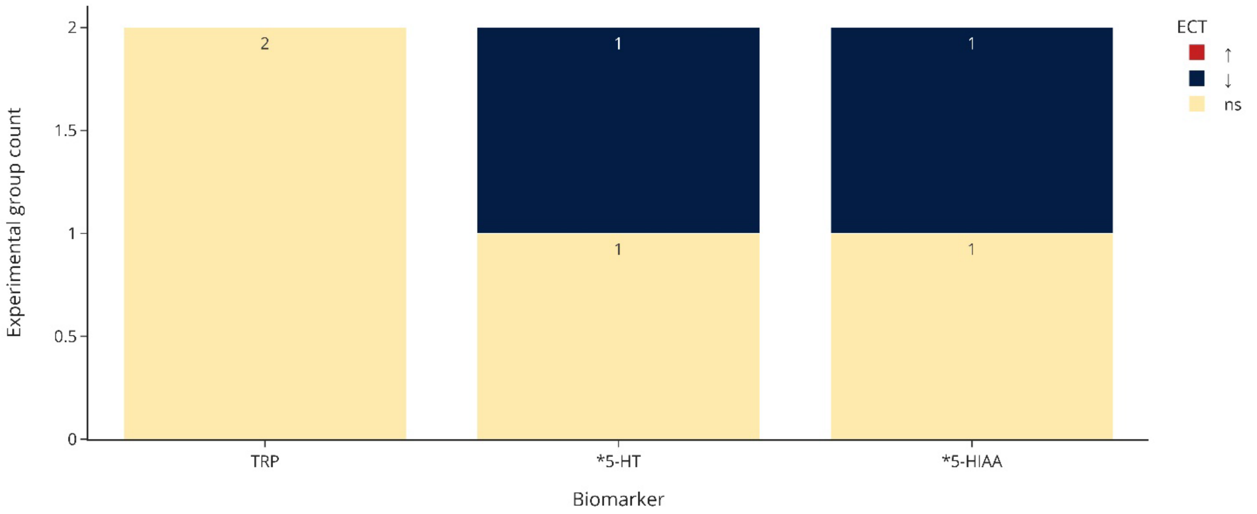

(e) Brain tissue (striatum) in preclinical studies

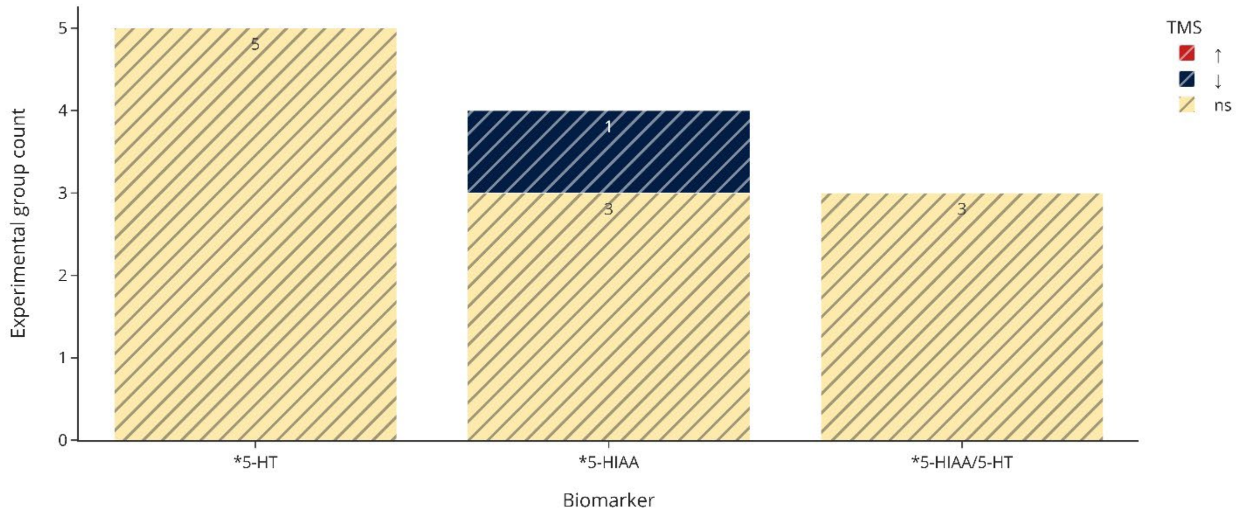

(f) Brain tissue (pineal gland) in preclinical studies

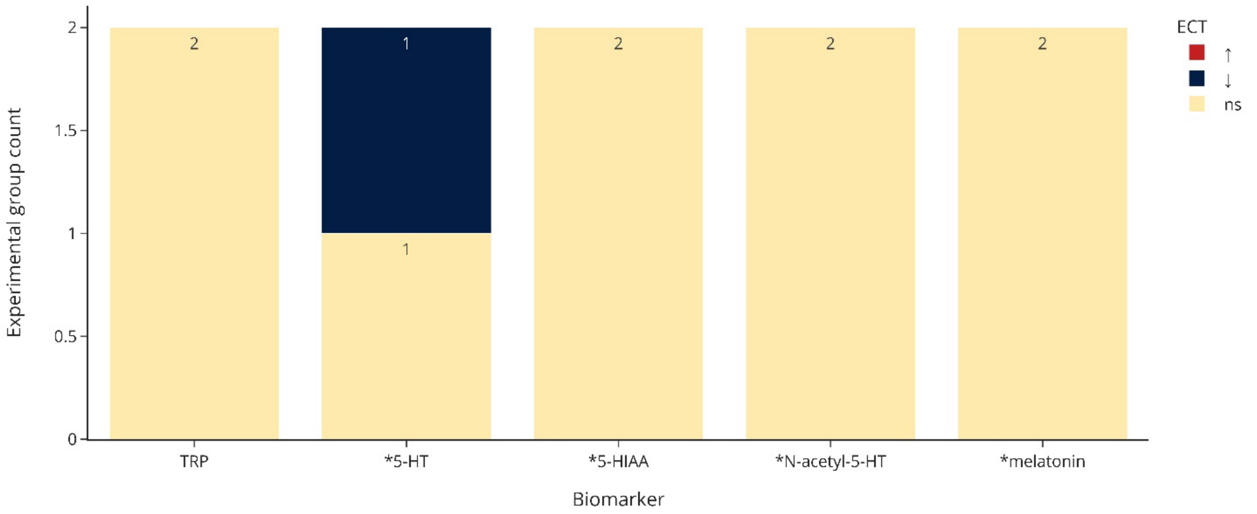

(g) Brain tissue (midbrain) in preclinical studies

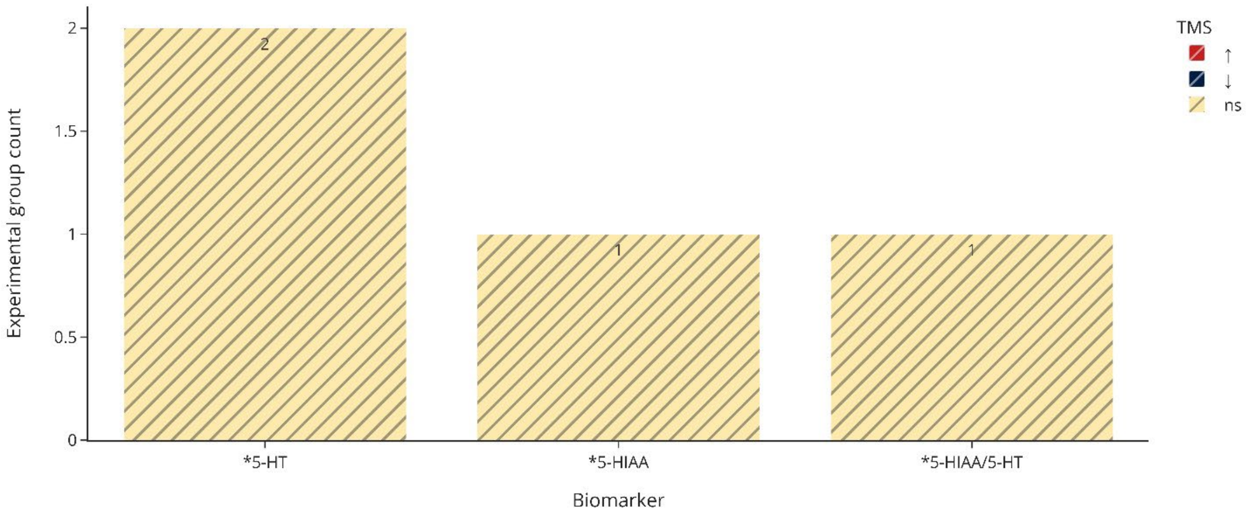

(h) Brain tissue (unspecified) in preclinical studies

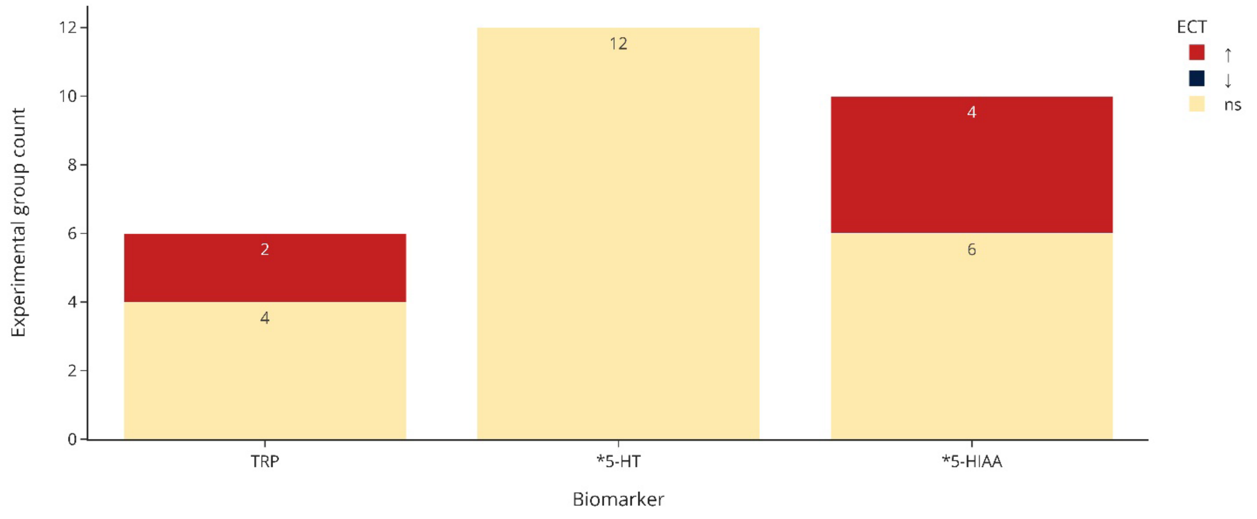

(i) Microdialysis (cortex) in preclinical studies

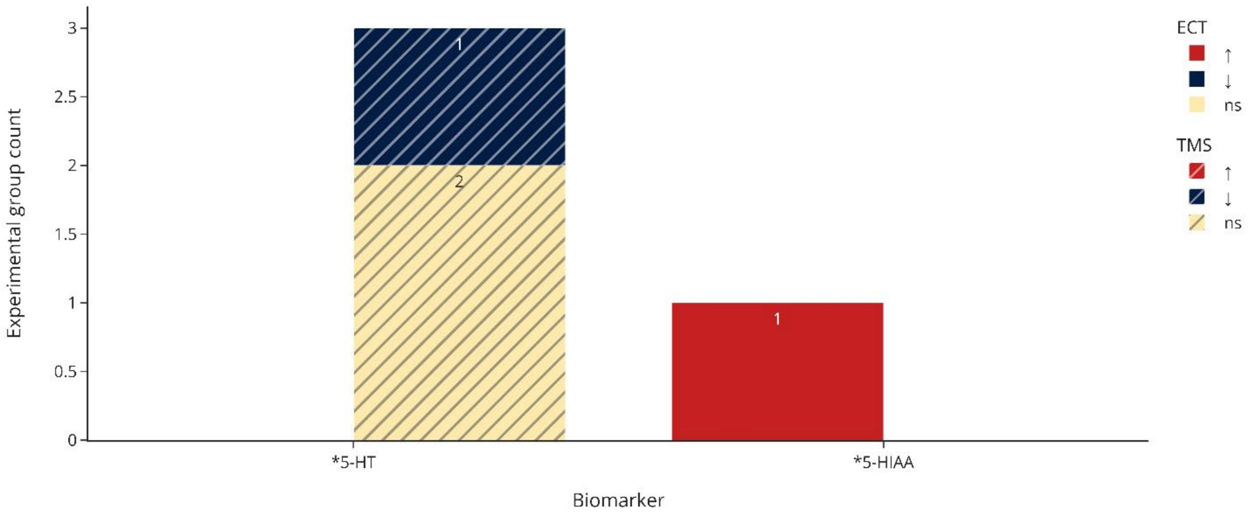

(j) Microdialysis (amygdala) in preclinical studies

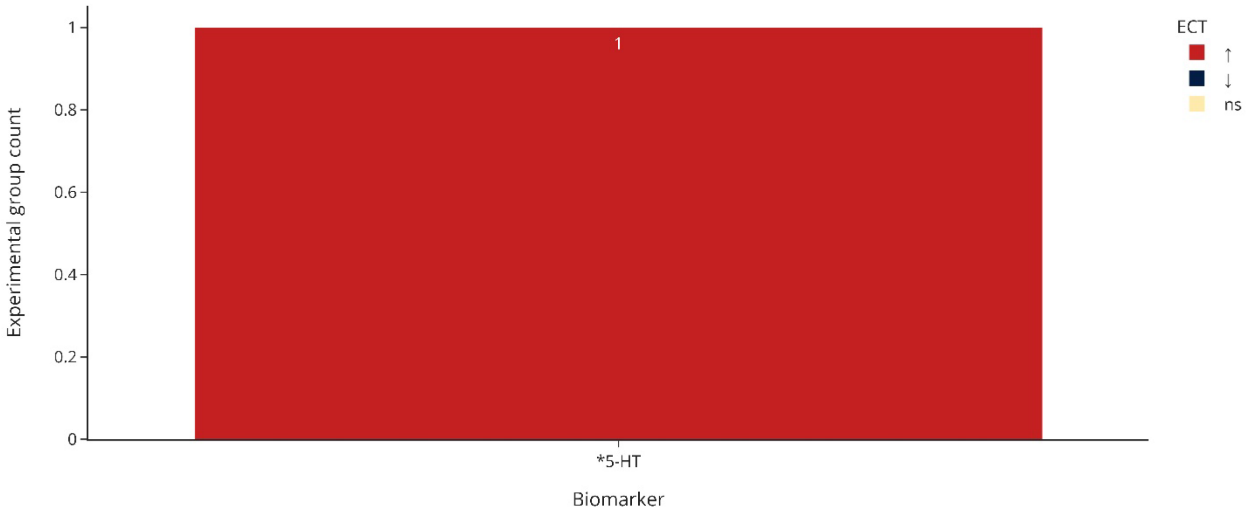

(k) Microdialysis (hippocampus) in preclinical studies

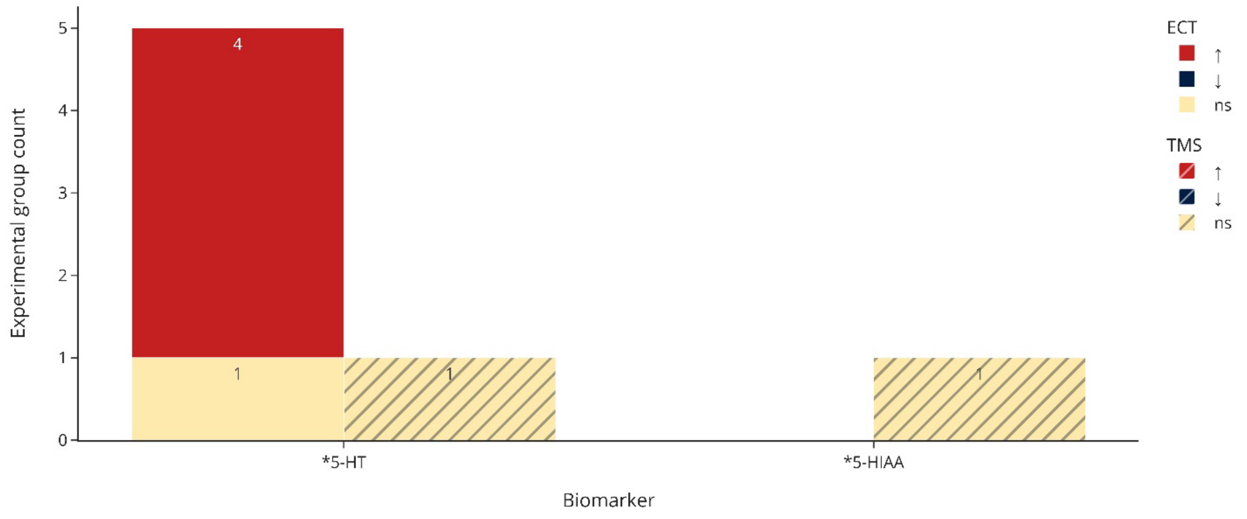

(l) Microdialysis (hypothalamus) in preclinical studies

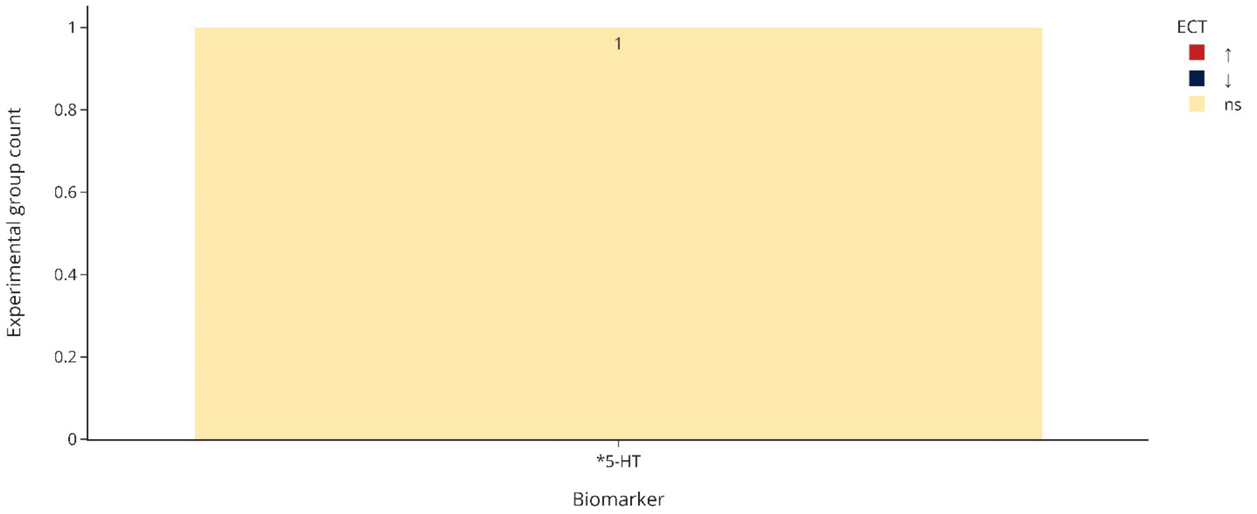

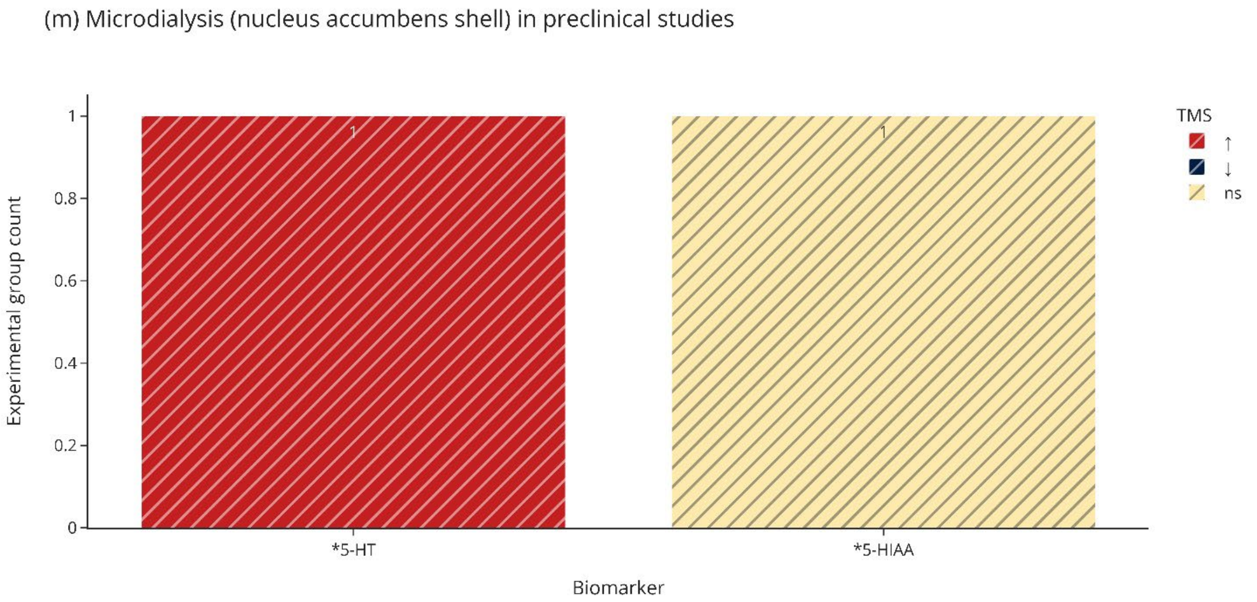

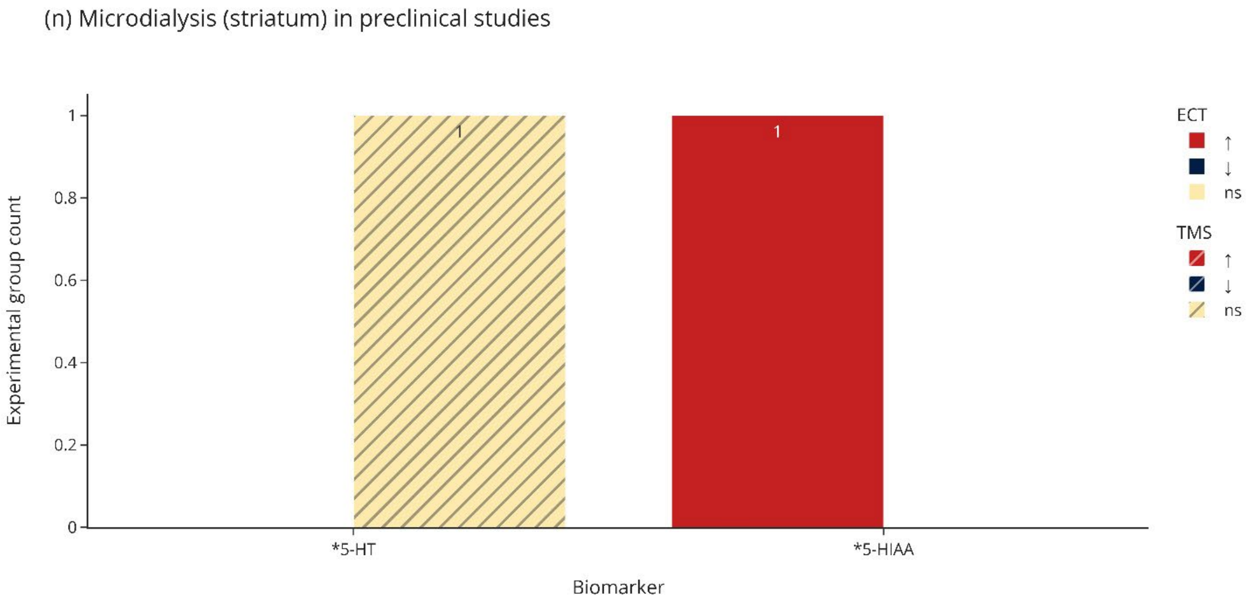

Figure S1. Count of experimental groups reporting significant increases, decreases, or non-significant changes in the levels of the biomarkers by non-invasive brain stimulation. (a-h) and (i-n) from the individual regions of brain tissue and microdialysis in preclinical studies, respectively. *: biomarkers in the serotonin pathway. Tryptophan refers to total tryptophan when not specified.
