# Supplementary material for: Non-Invasive Brain Stimulation Effects on Biomarkers of Tryptophan Metabolism: A Scoping Review and Meta-Analysis
Source: Int J Mol Sci. 2022 Aug 26;23(17):9692. doi: 10.3390/ijms23179692 (PMC9456364; doi:10.3390/ijms23179692)
Supplement: Supplementary file 1 [file ijms-23-09692-s001.zip › Figure S2 - Sources of biomarkers.pdf]

(a) Clinical studies

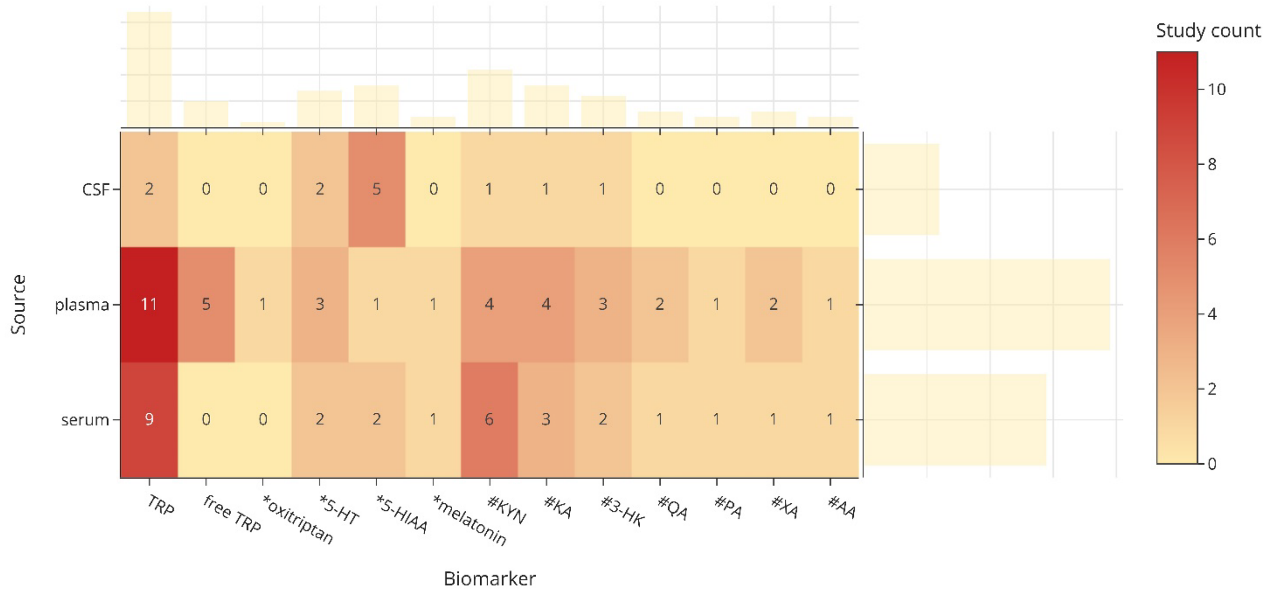

(b) Preclinical studies (brain regions collapsed)

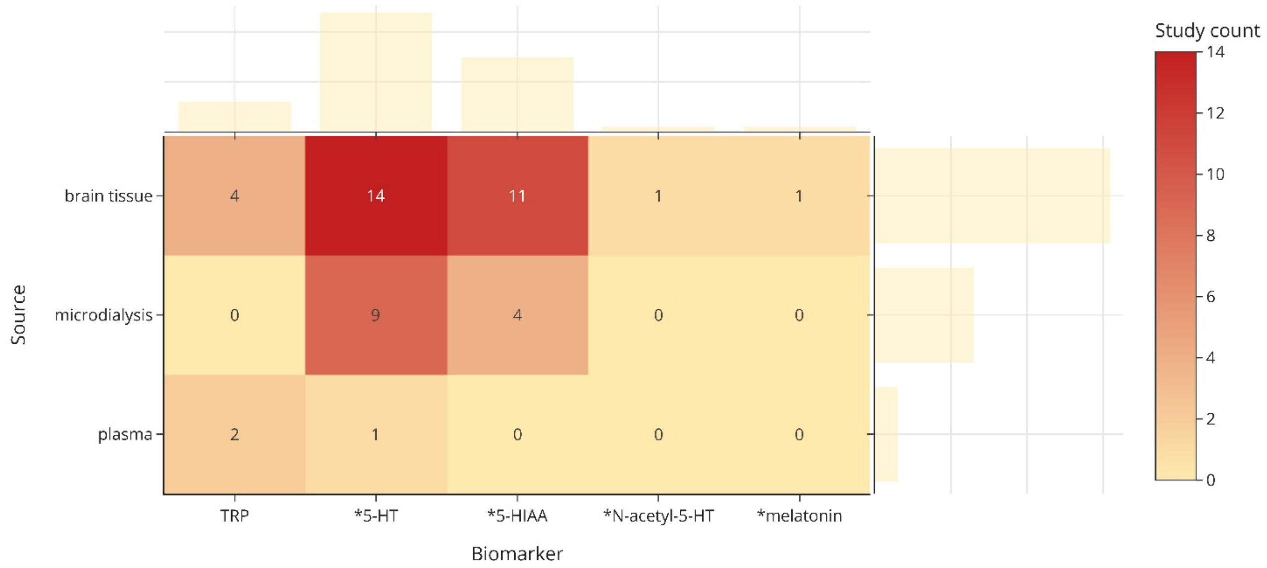

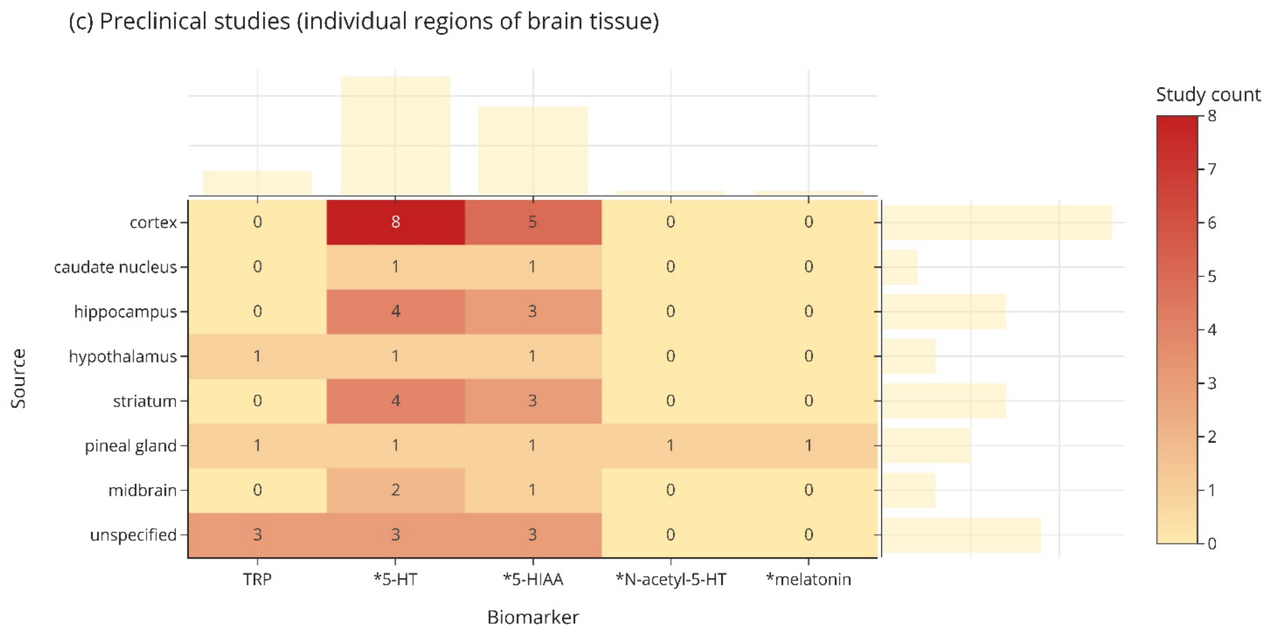

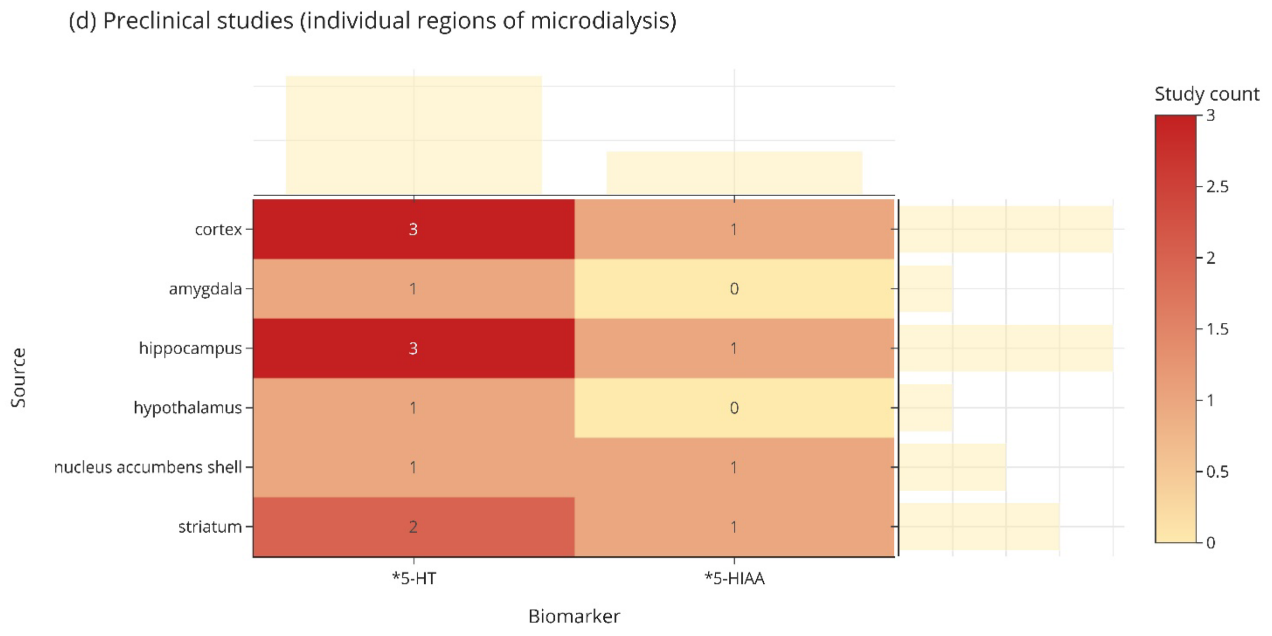

Figure S2. Sources of biomarkers in clinical studies (a) and preclinical studies, when the brain regions are collapsed (b) or shown individually (c-d). *: biomarkers in the serotonin pathway; #: biomarkers in the kynurenine pathway. Here TRP refers to total TRP when unspecified.
