# Supplementary material for: Non-Invasive Brain Stimulation Effects on Biomarkers of Tryptophan Metabolism: A Scoping Review and Meta-Analysis
Source: Int J Mol Sci. 2022 Aug 26;23(17):9692. doi: 10.3390/ijms23179692 (PMC9456364; doi:10.3390/ijms23179692)
Supplement: Supplementary file 1 [file ijms-23-09692-s001.zip › Supplementary Text.pdf]

Characteristics of Sources of Evidence and Individual Results

38 included studies recruited humans (including the case reports [1], Table 2), while 27 were preclinical studies (Table 3). The tables distinguish study designs, list the NIBS protocols, describe the features of the participants or subjects, point out the sources and timing of sampling, and cite the changes (or group differences) in the levels of the biomarkers. Additionally, clinical measures for the human participants were available in the Supplement Spreadsheets.

Of the 37 clinical studies (excluding the case reports [1]), 25 (68%) used ECT, and 11 (30%) used TMS. A lower percentage of the preclinical studies (12 of 27, 44%) employed ECT, while more (14, 52%) used TMS. There was one clinical and one preclinical tDCS study. A histogram of the study count versus publication years (Figure 3) shows the ECT studies, both clinical and preclinical, dominated the 1970s and 80s. The years around 2000 saw the emergence of preclinical TMS studies followed by the clinical ones. In the last decade, there has been no preclinical ECT study, yet the clinical ECT and TMS studies of both phases have been growing, plus the appearance of tDCS studies.

The clinical and preclinical studies showed similar and different interests in the biomarkers and sources of sampling, as illustrated in Figure S2. Both frequently investigated TRP and the pair of 5-HT and 5-HIAA. Whereas a visible proportion of the clinical studies looked into biomarkers in the kynurenine pathway, notably the pair of KYN and KA, kynurenine biomarkers were completely missing from the preclinical studies. In the clinical studies, the samples were exclusively from CSF or blood, including plasma and serum. In contrast, in the preclinical studies they were chiefly from brain tissue or collected by microdialysis, yet the specific regions varied. In two preclinical studies the samples were from plasma, but in none from serum. There were another three other peripheral sources each mentioned in one preclinical study only, thus excluded from the syntheses (myocardium tissue in [2], urine in [3], and ventral horn sections of the lumbar spinal cord in [4]).

Critical Appraisal within Sources of Evidence

We consider the clinical studies generally of inferior quality, due to the lack of randomized control designs compounded by pharmacotherapy-as-usual, though these compromises might be necessary. Of the 37 clinical studies (excluding the case reports [1]), 31 (84%) used the one-group pretest-posttest design, while six (16%) employed the parallel or crossover design, of which four were randomized, and two were not. The ECT participants were primarily referred to the treatment given the severity of their condition, and some had been scheduled for one before the studies [5, 6]. There was only one ECT study with a control group, yet not as a result of randomization but because they were not prescribed ECT owing to contraindications [7]. Many ECT and TMS studies mention the participants did not stop prior medication, and some cite ethical and practical reasons [6, 8, 9]. Regarding the preclinical studies, 25 of the 27 (93%) used the parallel design, and there was no pharmacotherapy-as-usual.

Additionally, we are concerned about the possible publication bias in both clinical and preclinical studies. We noticed 27 of the 37 clinical studies (73%) and 22 of the 27 preclinical studies (81%) also investigated molecules other than tryptophan metabolites, notably the pair of dopamine and homovanillic acid [12-15]. Given that only four included studies were preregistered [7, 16-18], chances are a considerable number of studies did not report nonsignificant changes in tryptophan metabolites, especially when there were significant changes in other molecules.

The heterogeneity of the NIBS protocols was examined as follows. A major distinction across NIBS types was between single and multiple sessions: among the clinical studies, 20 of the 25 using ECT (80%) entailed reportedly a maximum of 20 sessions, while five (20%) involved only one session only; all but one TMS study were multi-session. Among the preclinical studies, seven of the 11 using ECT (64%) were multi-session, two (18%) were single-session, and three (27%) could be either, depending on the experimental

group; eight of the 14 TMS studies (57%) were multi-session, while six (43%) were single-session. A major distinction within TMS was between high and low pulse frequency: four of the 11 clinical TMS studies (36%) used high frequency (10–20 Hz), six (55%) used low frequency (0.5–8 Hz), one (9%) employed both, depending on the experimental group; all preclinical studies used high frequency (5–25 Hz), though one also applied low frequency (1 Hz) to an experimental group. Other variations existed yet were supposed to make limited impacts.

The participants or subjects were largely homogeneous within the clinical and preclinical studies each, but highly different between the two phases. In most clinical studies (30 of 37, 81%), the participants were depressed patients (30 of 37, 81%; including those with treatment-resistant depression, TRD). One study each recruited patients with post-stroke depression [19], stroke [20], schizophrenia [21], generalised anxiety disorders [22], fibromyalgia [23], and Parkinson's disease [16], plus one study with healthy volunteers [24]. Of the 30 depression studies, 13 (43%) mention treatment resistance; 11 (of the 30, 37%) specify the unipolar form, 14 (47%) state there were both patients with the uni- and bipolar forms, and 15 (50%) provide no such information; one (3%) specifies that the patients exhibited psychotic symptoms, two (7%) specify the absence of psychotic symptoms, nine (30%) state there were both patients with and without psychotic symptoms, and 18 (60%) contain no such information. Regarding the demographics, the majority of the clinical studies recruited more females than males (25 of the 35 providing the gender ratio, 71%) or equally (two, 6%), and the age mean or median mainly fell between 40 and 60 (27 of the 36 offering such information, 72%). In contrast, most preclinical studies used healthy animals (22 of 27, 81%). Of all 27 studies, 24 (89%) used rats, two (7%) mice, and one (4%) dogs. In all but two studies providing the sex ratio, the animals were all males.

In many studies, the timing of post-NIBS sampling appeared arbitrarily chosen or is vaguely described. We attempted to classify the time points into the following four scales: a) the scale of minutes if during the NIBS session or earlier than one hour after the last session (one hour excluded); b) the scale of hours if later than one hour (included) yet on the same day; c) the scale of days, if between the next day and a week (excluded); d) the scale of weeks, if later than a week (included). Of the 25 clinical studies providing the timing relative to the last session, eight (32%), seven (28%), eight (32%), and 10 (40%) entailed time points on the scales of minutes, hours, days, and weeks, respectively. Similarly, of the 25 preclinical studies providing the timing, 11 (44%), 13 (52%), nine (36%), two (8%) involved time points on the scales of minutes, hours, days, and weeks, respectively. Note that some studies involved multiple or variable time points and hence were counted more than once across the scales.

Qualitative Analysis

Figures 5a-f are the series of visualised results consisting of the counts of experimental groups with significant or nonsignificant changes in the levels of the biomarkers. Figures 5a-c are for samples from CSF (a), plasma (b), and serum (c) in the clinical studies, and Figures 5d-f are for brain tissue (d), microdialysis (e), and plasma (f) in the preclinical studies. Here for brain tissue and microdialysis the regions are collapsed, but they are shown individually in Figure S1. For simplicity, only ECT and TMS results are visualised, and readers are referred to the bottom of Tables 2 or 3 for the only clinical or preclinical tDCS study, respectively.

There was one case of conflicting results in [25], where the plasma level of free TRP was significantly higher than the baseline at 1 min post-ECT, nonsignificantly different from the baseline between 5 and 30 min post-ECT, but significantly lower at 1 h post-ECT. It is worth noting that in a similar study (also single-session and recruiting depressed patients) [26], the plasma level of free TRP was significantly higher at 1 and 15 min post-ECT, and while it was nonsignificantly different from the baseline at 1 h post-ECT, its mean was lower than the baseline mean.

Regarding clinical studies, we observed heterogeneity in the plasma level of TRP affected by ECT as there were experimental groups counted for significant changes in opposite directions (the column in red and blue in Figure 5b). All groups involved were depressed patients, and the majority underwent multiple sessions. However, the timing of post-ECT sampling varied greatly: the only group counted for increases revealed the level rose at 2, 6, and 24 h but returned to the baseline at 48 h [27]. Two groups counted for decreases showed the level fell at 10 min [25, 28], but one of them showed it returned to the baseline at 30 min [25]. The other two groups counted for decreases did not provide the timing [9, 29]. The four groups counted for nonsignificant changes were sampled across the scales of minutes, hours (1 min–1 hour [26]), and days (1–7 days [30-32]).

The only sign of effects was detected in the serum level of 5-HT significantly increased by TMS in two experimental groups (Figure 5c). Both underwent multiple sessions of low-frequency TMS. However, one comprised patients with post-stroke depression and were sampled eight weeks after the last session [19], while the other consisted of those with generalised anxiety disorders and were sampled at 1 h post-TMS [22]. Any other significant change either had not been repeated (for example, the CSF level of 5-HT affected by ECT, Figure 5a) or was less evidenced than the nonsignificant counterpart according to the experimental group count (for instance, the CSF level of 5-HIAA affected by ECT, the Figure 5a).

Regarding preclinical studies, we perceived that the effect of ECT and TMS on the pair of 5-HT and 5-HIAA in the brain might depend on the specific regions. In the bar chart for brain tissue collapsing the regions, in the 5-HT level affected by ECT and the 5-HIAA level affected by ECT or TMS, there were experimental groups counted for significant changes in opposite directions (the columns in red and blue in Figure 5d). However, we noticed the significant change in each direction was attributed to different regions. For example, the experimental group with the 5-HT level increased by ECT had its sample collected from the cortex [3], while the two groups with the reduced level had their samples taken from the hypothalamus or pineal gland [33]. The same applies to the microdialysated level of 5-HT affected by TMS (the columns in red and blue in Figure 5e). When we examined the bar charts for the individual regions, whether brain tissue or microdialysis, we found no sign of heterogeneity (Figure S1).

We found no indication of effects in brain tissue, microdialysis, or plasma, except in the microdialysated level of 5-HT affected by ECT, the five counts for significant increases outweigh the two counts for nonsignificant changes (Figure 5e). However, the five experimental groups counted for increases were derived from the same study trying various sites of stimulation on anaesthetised or behaving rats [34]. Therefore, these changes are not considered reproduced, hence not an indication of effects.

In comparison, all studies reporting clinical measures had at least one measure significantly improved by NIBS (data available in the Supplement Spreadsheets).

References

1. Rudorfer, M.V., et al., *Disparate Biochemical Actions of Electroconvulsive Therapy and Antidepressant Drugs.* Convuls Ther, 1988. **4**(2): p. 133-140.
2. Khanna, N.K., P. Lauria, and V.N. Sharma, *5-hydroxytryptamine content of the dog myocardium after chronic electroconvulsive therapy.* Indian J Physiol Pharmacol, 1971. **15**(4): p. 187-8.
3. Karoum, F., et al., *The effects of desipramine, zimelidine, electroconvulsive treatment and lithium on rat brain biogenic amines: a comparison with peripheral changes.* Eur J Pharmacol, 1986. **121**(3): p. 377-85.
4. Wang, S., et al., *Combination of repetitive transcranial magnetic stimulation and treadmill training reduces hyperreflexia by rebalancing motoneuron excitability in rats after spinal cord contusion.* Neurosci Lett, 2022. **775**: p. 136536.
5. Nikisch, G. and A.A. Mathe, *CSF monoamine metabolites and neuropeptides in depressed patients before and after electroconvulsive therapy.* Eur Psychiatry, 2008. **23**(5): p. 356-9.

6. Smith, D.F. and L.S. Stromgren, *Influence of unilateral ECT on tryptophan metabolism in endogenous depression.* Pharmacopsychiatria, 1981. **14**(4): p. 135-8.
7. Hasani, P.A.M., et al., *Effect of Electroconvulsive Therapy on Serum Serotonin Level in Patients with Treatment- Resistant Major Depressive Disorder.* Journal of Evolution of Medical and Dental Sciences, 2019. **8**(16): p. 1283-1286.
8. Olajossy, M., et al., *Blood serum concentrations of kynurenic acid in patients diagnosed with recurrent depressive disorder, depression in bipolar disorder, and schizoaffective disorder treated with electroconvulsive therapy.* Psychiatr Pol, 2017. **51**(3): p. 455-468.
9. Schwieler, L., et al., *Electroconvulsive therapy suppresses the neurotoxic branch of the kynurenine pathway in treatment-resistant depressed patients.* J Neuroinflammation, 2016. **13**(1): p. 51.
10. Lefaucheur, J.P., et al., *Evidence-based guidelines on the therapeutic use of repetitive transcranial magnetic stimulation (rTMS): An update (2014-2018).* Clin Neurophysiol, 2020. **131**(2): p. 474-528.
11. McGrath, S., et al., *Estimating the sample mean and standard deviation from commonly reported quantiles in meta-analysis.* Stat Methods Med Res, 2020: p. 962280219889080.
12. Ben-Shachar, D., et al., *Transcranial magnetic stimulation induces alterations in brain monoamines.* J Neural Transm (Vienna), 1997. **104**(2-3): p. 191-7.
13. Ben-Shachar, D., et al., *Chronic repetitive transcranial magnetic stimulation alters beta-adrenergic and 5-HT2 receptor characteristics in rat brain.* Brain Res, 1999. **816**(1): p. 78-83.
14. Miniussi, C., et al., *Repetitive transcranial magnetic stimulation (rTMS) at high and low frequency: an efficacious therapy for major drug-resistant depression?* Clin Neurophysiol, 2005. **116**(5): p. 1062-71.
15. Keck, M.E., et al., *Acute transcranial magnetic stimulation of frontal brain regions selectively modulates the release of vasopressin, biogenic amines and amino acids in the rat brain.* Eur J Neurosci, 2000. **12**(10): p. 3713-20.
16. Hadoush, H., et al., *Melatonin serum level, sleep functions, and depression level after bilateral anodal transcranial direct current stimulation in patients with Parkinson's disease: a feasibility study.* Sleep Sci, 2021. **14**(Spec 1): p. 25-30.
17. Tateishi, H., et al., *The changes in kynurenine metabolites induced by rTMS in treatment-resistant depression: A pilot study.* J Psychiatr Res, 2021. **138**: p. 194-199.
18. Tateishi, H., et al., *Changes in the metabolites of cerebrospinal fluid induced by rTMS in treatment-resistant depression: A pilot study.* Psychiatry Res, 2022. **313**: p. 114636.
19. Liu, S., et al., *Effect of transcranial magnetic stimulation on treatment effect and immune function.* Saudi J Biol Sci, 2022. **29**(1): p. 379-384.
20. Niimi, M., et al., *Effect of repetitive transcranial magnetic stimulation on the kynurenine pathway in stroke patients.* Neuroreport, 2020. **31**(9): p. 629-636.
21. Udayakumar, M.A., et al., *Influence of single ECT on dopamine, noradrenaline and 5-hydroxytryptamine concentrations in CSF of schizophenics.* The Indian Journal of Medical Research, 1981. **74**(5): p. 757-762.
22. Lu, R., et al., *The effect of bilateral low-frequency rTMS over dorsolateral prefrontal cortex on serum brain-derived neurotropic factor and serotonin in patients with generalized anxiety disorder.* Neurosci Lett, 2018. **684**: p. 67-71.
23. Maestu, C., et al., *Reduction of pain thresholds in fibromyalgia after very low-intensity magnetic stimulation: a double-blinded, randomized placebo-controlled clinical trial.* Pain Res Manag, 2013. **18**(6): p. e101-6.
24. Sibon, I., et al., *Acute prefrontal cortex TMS in healthy volunteers: effects on brain 11C-alphaMtrp trapping.* Neuroimage, 2007. **34**(4): p. 1658-64.
25. Sawa, Y., *The effect of electroconvulsive therapy on plasma cyclic-AMP, non-esterified fatty acid, tryptophan and tyrosine in depression.* Keio J Med, 1981. **30**(4): p. 193-204.
26. Stelmasiak, Z. and G. Curzon, *Effect of electroconvulsive therapy on plasma unesterified fatty acid and free tryptophan concentrations in man.* J Neurochem, 1974. **22**(4): p. 603-4.
27. Palmio, J., et al., *Changes in plasma amino acids after electroconvulsive therapy of depressed patients.* Psychiatry Res, 2005. **137**(3): p. 183-90.

28. Whalley, L.J., C.M. Yates, and J.E. Christie, *Effect of electroconvulsive therapy (ECT) on plasma tryptophan.* Psychol Med, 1980. **10**(2): p. 377-80.
29. Kirkegaard, C., S.E. Moller, and N. Bjorum, *Addition of L-tryptophan to electroconvulsive treatment in endogenous depression. A double-blind study.* Acta Psychiatr Scand, 1978. **58**(5): p. 457-62.
30. Allen, A.P., et al., *Kynurenine pathway metabolism and the neurobiology of treatment-resistant depression: Comparison of multiple ketamine infusions and electroconvulsive therapy.* J Psychiatr Res, 2018. **100**: p. 24-32.
31. Hoekstra, R., et al., *Effect of electroconvulsive therapy on biopterin and large neutral amino acids in severe, medication-resistant depression.* Psychiatry Res, 2001. **103**(2-3): p. 115-23.
32. Ryan, K.M., et al., *Tryptophan metabolite concentrations in depressed patients before and after electroconvulsive therapy.* Brain Behav Immun, 2020. **83**: p. 153-162.
33. McIntyre, I.M. and G.F. Oxenkrug, *Electroconvulsive shock: effect on pineal and hypothalamic indoles.* J Pineal Res, 1984. **1**(3): p. 273-9.
34. Juckel, G., A. Mendlin, and B.L. Jacobs, *Electrical stimulation of rat medial prefrontal cortex enhances forebrain serotonin output: implications for electroconvulsive therapy and transcranial magnetic stimulation in depression.* Neuropsychopharmacology, 1999. **21**(3): p. 391-8.
