# Supplementary material for: Non-Invasive Brain Stimulation Effects on Biomarkers of Tryptophan Metabolism: A Scoping Review and Meta-Analysis
Source: Int J Mol Sci. 2022 Aug 26;23(17):9692. doi: 10.3390/ijms23179692 (PMC9456364; doi:10.3390/ijms23179692)
Supplement: Supplementary file 1 [file ijms-23-09692-s001.zip › Table S1 - Search queries.pdf]

Table S2. Search queries for PubMed and Web of Science databases.

**PubMed search query**
("magnetic stimulation"[Title/Abstract] OR TMS[Title/Abstract] OR rTMS[Title/Abstract] OR "electroconvulsive therapy"[Title/Abstract] OR ECT[Title/Abstract] OR "theta burst stimulation"[Title/Abstract] OR TBS[Title/Abstract] OR "transcranial Direct Current Stimulation"[Title/Abstract] OR tDCS[Title/Abstract]) AND ((tryptophan[Title/Abstract] OR kynurenine[Title/Abstract] OR kynurenic[Title/Abstract] OR formylkynurenine[Title/Abstract] OR quinolinic[Title/Abstract] OR "NAD+"[Title/Abstract] OR 3-OH-kynurenine[Title/Abstract] OR xanthurenic[Title/Abstract] OR picolinic[Title/Abstract] OR anthranilic[Title/Abstract] OR 5-hydroxytryptophan[Title/Abstract] OR oxitriptan[Title/Abstract] OR 5-hydroxytryptamine[Title/Abstract] OR 5-Hydroxyindoleacetic[Title/Abstract] OR N-acetyl-5-HT[Title/Abstract] OR melatonin[Title/Abstract]) OR ((5-HT[Title/Abstract] OR serotonin[Title/Abstract]) AND (level*[Title/Abstract] OR serum[Title/Abstract] OR plasma[Title/Abstract] OR "cerebrospinal fluid"[Title/Abstract] OR CSF[Title/Abstract] OR microdialysis[Title/Abstract])))
**Web of Science search query**
[Title/Abstract] ("magnetic stimulation" OR TMS OR rTMS OR "electroconvulsive therapy" OR ECT OR "theta burst stimulation" OR TBS OR "transcranial Direct Current Stimulation" OR tDCS) AND ((tryptophan OR kynurenine OR kynurenic OR formylkynurenine OR quinolinic OR "NAD+" OR 3-OH-kynurenine OR xanthurenic OR picolinic OR anthranilic OR 5-hydroxytryptophan OR oxitriptan OR 5-hydroxytryptamine OR 5-Hydroxyindoleacetic OR N-acetyl-5-HT OR melatonin) OR ((5-HT OR serotonin) AND (level* OR serum OR plasma OR "cerebrospinal fluid" OR CSF OR microdialysis)))
