# Supplementary material for: Non-Invasive Brain Stimulation Effects on Biomarkers of Tryptophan Metabolism: A Scoping Review and Meta-Analysis
Source: Int J Mol Sci. 2022 Aug 26;23(17):9692. doi: 10.3390/ijms23179692 (PMC9456364; doi:10.3390/ijms23179692)
Supplement: Supplementary file 1 [file ijms-23-09692-s001.zip › Table S2 - Excluded studies.pdf]

International Journal of
Molecular Sciences

MDPI

Table S2. Studies excluded during full-text screening and reasons, or studies that could not be retrieved.

Study	Reason(s) for exclusion
Abrams et al., 1976 [1]	Could not retrieve.
Belmaker & Grisaru, 1998 [2]	Report same data in [3].
Cassidy et al., 1997 [4]	Assessed biomarker response to tryptophan depletion after ECT series.
Cassidy et al., 2010 [5]	Assessed biomarker response to tryptophan depletion after ECT series.
Chang et al., 2011 [6]	Biomarkers of interest not assessed.
Costain et al., 1978 [7]	Not an experiment.
d'Elia et al., 1979 [8]	Reported same data in [9].
d'Elia et al., 1977 [10]	Biomarkers of interest not assessed.
d'Elia et al., 1978 [11]	ECT effects on biomarkers not reported (although authors reported collecting).
Dalal et al., 1997 [12]	Kinetics study, not assessing biomarker levels.
Green, 1978 [13]	Not a NIBS experiment.
Ikeda et al., 2005 [14]	Kinetics study, not assessing biomarker levels.
Krahn et al., 2000 [15]	Biomarkers of interest not assessed.
Kranaster et al., 2020 [16]	Post-ECT biomarkers not assessed.
Liu et al., 2020 [17]	Not a NIBS experiment.
Mano et al., 1989 [18]	Could not retrieve.
Molnar et al., 1979 [19]	Biomarkers of interest not assessed.
Mohamad Safiai et al., 2020 [20]	Protocol, no results reported.
Nordin et al., 1971 [21]	Merged ECT effects with flurothyl convulsive therapy.
Olajossy et al., 2018 [22]	Reporting same data in [23].
Papakostas et al., 1990 [24]	Biomarkers of interest not assessed.
Papakostas et al., 2000 [25]	Biomarkers of interest not assessed.
Peng et al., 2021 [26]	Biomarker sampling method is dubious.
Potter et al., 1987 [27]	Review.
Price et al., 1985 [28]	Biomarkers of interest not assessed.
Rausch et al., 1988 [29]	Platelet uptake study, not assessing biomarker levels.
Shiiba et al., 2012 [30]	Invasive, stimulated mice sciatic nerve of directly.
Wagner et al., 1987 [31]	Reported same data [32]
Wang et al., 2004 [33]	Biomarkers of interest not assessed.

References

1. Abrams, R., et al., *Concentration of 5-hydroxyindoleacetic acid, homovanillic acid, and tryptophan in the cerebrospinal fluid of depressed patients before and after ECT.* Biological Psychiatry, 1976. **11**(1): p. 85-90.
2. Belmaker, R.H. and N. Grisaru, *Magnetic stimulation of the brain in animal depression models responsive to ECS.* J ECT, 1998. **14**(3): p. 194-205.

3. Ben-Shachar, D., et al., *Transcranial magnetic stimulation induces alterations in brain monoamines.* J Neural Transm (Vienna), 1997. **104**(2-3): p. 191-7.
4. Cassidy, F., et al., *Lack of Relapse With Tryptophan Depletion Following Successful Treatment With ECT.* The American Journal of Psychiatry, 1997. **154**(8): p. 1151-1152.
5. Cassidy, F., et al., *Combined catecholamine and indoleamine depletion following response to ECT.* Br J Psychiatry, 2010. **196**(6): p. 493-4.
6. Chang, T.G., et al., *Application of electroconvulsive therapy in treatment of retinitis pigmentosa comorbid with major depressive disorder and panic disorder.* J ECT, 2011. **27**(4): p. e57-8.
7. Costain, D.W., D.G. Grahame-Smith, and A.R. Green, *Relevance of the enhanced 5-hydroxytryptamine behavioural responses in rats to electroconvulsive therapy.* British Journal of Pharmacology, 1978. **62**(3): p. 394P.
8. d'Elia, G., J. Lehmann, and H. Raotma, *Bimodal distribution of serum trypotphan level.* Acta Psychiatr Scand, 1979. **60**(1): p. 10-6.
9. d'Elia, G., J. Lehmann, and H. Raotma, *Evaluation of the combination of tryptophan and ECT in the treatment of depression.* Acta Psychiatrica Scandinavica, 1977. **56**(4): p. 319-334.
10. d'Elia, G., J. Lehmann, and H. Raotma, *Evaluation of the combination of tryptophan and ECT in the treatment of depression.* Acta Psychiatrica Scandinavica, 1977. **56**(4): p. 303-318.
11. d'Elia, G., J. Lehmann, and H. Raotma, *Influence of tryptophan on memory functions in depressive patients treated with unilateral ECT.* Acta Psychiatr Scand, 1978. **57**(3): p. 259-68.
12. Dalal, P.K., et al., *Ect and platelet 5ht uptake in major depression.* Indian J Psychiatry, 1997. **39**(4): p. 272-7.
13. Green, A.R., *Repeated exposure of rats to the convulsant agent flurothyl enhances 5-hydroxytryptamine- and dopamine-mediated behavioural responses.* British Journal of Pharmacology, 1978. **62**(3): p. 325-331.
14. Ikeda, T., et al., *Modulation of monoamine transporter expression and function by repetitive transcranial magnetic stimulation.* Biochem Biophys Res Commun, 2005. **327**(1): p. 218-24.
15. Krahn, L.E., et al., *The Effects of Electroconvulsive Therapy on Melatonin.* The Journal of ECT, 2000. **16**(4): p. 391-398.
16. Kranaster, L., et al., *The novel seizure quality index for the antidepressant outcome prediction in electroconvulsive therapy: association with biomarkers in the cerebrospinal fluid.* Eur Arch Psychiatry Clin Neurosci, 2020. **270**(7): p. 911-919.
17. Liu, H., et al., *Effects of repetitive magnetic stimulation on motor function and GAP43 and 5-HT expression in rats with spinal cord injury.* J Int Med Res, 2020. **48**(12): p. 300060520970765.
18. Mano, Y., et al., *[The kinesiological, chemical and pathological analysis in pulsed magnetic stimulation to the brain].* Rinsho Shinkeigaku, 1989. **29**(8): p. 982-8.
19. Molnar, L., I. Degrell, and S. Rochlitz, *[Effect of bilateral and unilateral electroconvulsive therapy (ECT) on the composition of the cerebrospinal fluid (CSF). A possibility to calculate the intracellular redox changes of the brain in humans (author's transl)].* Arch Psychiatr Nervenkr (1970), 1979. **227**(2): p. 159-69.
20. Mohamad Safiai, N.I., et al., *Effectiveness and tolerability of repetitive transcranial magnetic stimulation for preventive treatment of episodic migraine: a single-centre, randomised, double-blind, sham-controlled phase 2 trial (Magnet-EM).* Trials, 2020. **21**(1): p. 923.
21. Nordin, G., J.O. Ottosson, and B.E. Roos, *Influence of convulsive therapy on 5-hydroxyindoleacetic acid and homovanillic acid in cerebrospinal fluid in endogenous depression.* Psychopharmacologia, 1971. **20**(4): p. 315-20.
22. Olajossy, M., et al., *Differences in the dynamics of changes in the concentration of kynurenic acid in the blood serum of depressed patients treated with electroconvulsive therapy.* Psychiatr Danub, 2018. **30**(3): p. 331-339.

23. Olajossy, M., et al., *Blood serum concentrations of kynurenic acid in patients diagnosed with recurrent depressive disorder, depression in bipolar disorder, and schizoaffective disorder treated with electroconvulsive therapy.* Psychiatr Pol, 2017. **51**(3): p. 455-468.
24. Papakostas, Y., et al., *Thyrotropin and Prolactin Secretion During ECT: Implications for the Mechanism of ECT Action.* Convuls Ther, 1990. **6**(3): p. 214-220.
25. Papakostas, Y.G., et al., *Administration of Citalopram Before ECT: Seizure Duration and Hormone Responses.* The Journal of ECT, 2000. **16**(4): p. 356-360.
26. Peng, Y., et al., *The Clinical Efficacy and Possible Mechanism of Combination Treatment of Cerebral Ischemic Stroke with Ginkgo Biloba Extract and Low-Frequency Repetitive Transcranial Magnetic Stimulation.* Sichuan Da Xue Xue Bao Yi Xue Ban, 2021. **52**(5): p. 883-889.
27. Potter, W.Z., et al., *Effects of psychotropic drugs on neurotransmitters in man.* Life Sciences, 1987. **41**(7): p. 817-820.
28. Price, W.A. and B. Zimmer, *Effects of L-Tryptophan on Electroconvulsive Therapy Seizure Time.* The Journal of Nervous and Mental Disease, 1985. **175**(10): p. 636-638.
29. Rausch, J.L., C.L. Rich, and S.C. Risch, *Platelet serotonin transport after a single ECT.* Psychopharmacology (Berl), 1988. **95**(1): p. 139-41.
30. Shiiba, S.J., et al., *Cutaneous magnetic stimulation reduces rat chronic pain via activation of the supra-spinal descending pathway.* Cell Mol Neurobiol, 2012. **32**(2): p. 245-53.
31. Wagner, A., et al., *Effects of Antidepressant Treatments on Platelet Tritiated Imipramine Binding in Major Depressive Disorder.* Archives of General Psychiatry, 1987. **44**(10): p. 870-877.
32. Aberg-Wistedt, A., et al., *Electroconvulsive Therapy Effects on Cerebrospinal Fluid Monoamine Metabolites and Platelet Serotonin Uptake In Melancholia.* Convulsive Therapy, 1986. **2**(2): p. 91-98.
33. Wang, M., et al., *[Study on the influence of simulative EEG modulation magnetic field on the discharge of median raphe nuclei].* Sheng Wu Yi Xue Gong Cheng Xue Za Zhi, 2004. **21**(2): p. 219-24.
